# Experimental and Numerical Simulations of 3D-Printed Polycaprolactone Scaffolds for Bone Tissue Engineering Applications

**DOI:** 10.3390/ma14133546

**Published:** 2021-06-25

**Authors:** Zhanyan Xu, Abdalla M. Omar, Paulo Bartolo

**Affiliations:** School of Mechanical, Aerospace and Civil Engineering, University of Manchester, Manchester M13 9PL, UK; zhanyan.xu@postgrad.manchester.ac.uk (Z.X.); abdalla.omar@postgrad.manchester.ac.uk (A.M.O.)

**Keywords:** additive manufacturing, bone scaffolds, finite element analysis, mechanical analysis

## Abstract

Ideal bone scaffolds for tissue engineering should be highly porous allowing cell attachment, spreading, and differentiation and presenting appropriate biomechanical properties. These antagonistic characteristics usually require extensive experimental work to achieve optimised balanced properties. This paper presents a simulation approach to determine the mechanical behaviour of bone scaffolds allowing the compressive modulus and the deformation mechanisms to be predicted. Polycaprolactone scaffolds with regular square pores and different porosities were considered. Scaffolds were also printed using an extrusion-based additive manufacturing and assessed under compressive loads. Similar designs were used for both simulation and fabrication steps. A good correlation between numerical and experimental results was obtained, highlighting the suitability of the simulation tool for the mechanical design of 3D-printed bone scaffolds.

## 1. Introduction

The scaffold-based approach is the most relevant bone tissue engineering strategy [1,2,3]. In this approach, scaffolds are three-dimensional (3D) physical substrates designed to promote cell attachment, differentiation, and proliferation, promoting the formation of a new tissue [4,5]. Ideal bone scaffolds should be biocompatible, bioactive, biodegradable (the degradation rate of the scaffolds should be similar to the regeneration rate of the native tissue), and porous to enable cell seeding and vascularization (pore sizes ranging between 100 and 300 μm) and should present appropriate mechanical properties (e.g., for in vitro applications, scaffolds must provide space for cell spreading and extracellular matrix formation, whereas for in vivo applications, the scaffolds must provide support for tissue regeneration and structural integrity in the site of injury) [6,7,8,9].

**Figure 1:**
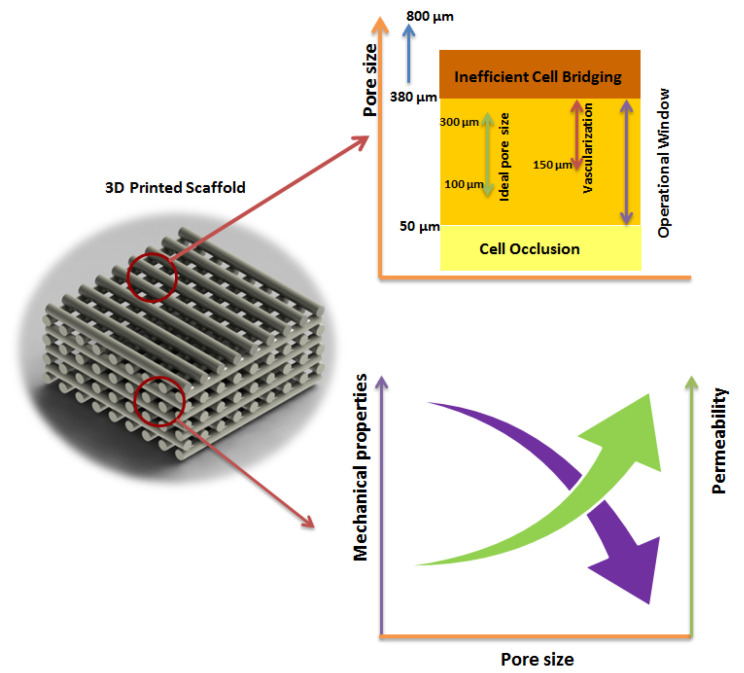
The effect of pore size on the mechanical and biological performance of printed scaffolds [14].

The design of pore size and, consequently, scaffold porosity is critical due its conflicting effect on the mechanical properties, cell seeding efficiency, cell attachment, and spreading and vascularisation (Figure 1). To minimise the extensive experimental work required to design bone scaffolds, different computational tools have been proposed. These tools are usually based on the use of analytical methods comprising empirical relationships between structural parameters and mechanical properties; CAD-based modelling methods, where the scaffold is designed using a repetition of 3D building blocks; CT-based methods using an image-based approach; and the homogenisation theory, which is a multilevel approach able to describe the scaffold at both the micro and macro levels [10].

**Figure 2:**
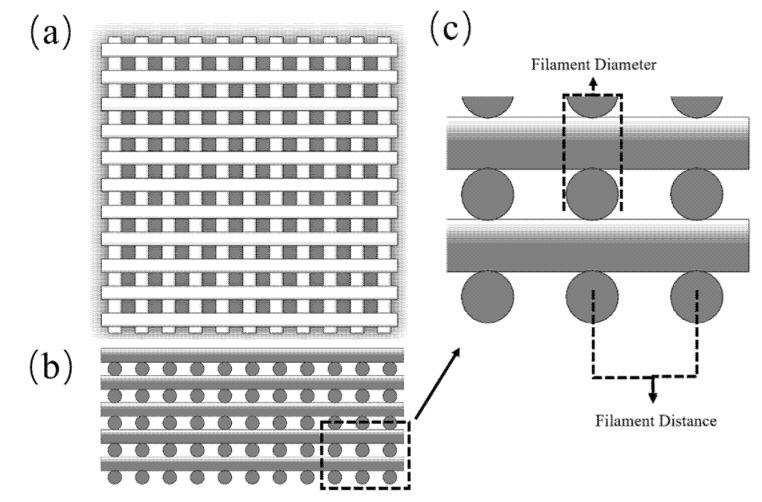
Scaffold configuration: (**a**) top view and (**b**) front view of the scaffold model. (**c**) Indication of filament distance and filament diameter.

This paper presents a simple and inexpensive strategy, validated against experimental results, considering (Figure 2):

- Linear elastic model: simulations are performed considering small deformations to guarantee the structural stability of the scaffolds. Once implanted, it is important that the porous channels remain stable to avoid any negative impact on cell spreading and nutrients and oxygen supply. Scaffolds should be designed avoiding plastic deformation or significant deformations.
- Materials are assumed to be homogeneous and isotropic.
- No separation contact condition between the scaffold filaments and the two compressive plates.
- Pore size differences between designed and printed scaffolds are neglected.

The simulations focused on polycaprolactone (PCL) scaffolds, widely investigated for bone tissue engineering applications, with uniformly distributed squared pores of different sizes [11,12,13,14].

## 2. Materials and Methods

### 2.1. Material

PCL pellets (CAPA^®^ 6500, Mw = 50,000 g/mol) with a size of 3 mm supplied by Perstorp (Warrington, UK) were used as received. The density of the PCL pellets is 1.1 g/cm^3^, the melting temperature is 58–60 °C, and the glass transition temperature is −60 °C.

### 2.2. Scaffold Design and Fabrication

Four groups of scaffolds were designed with a 0°/90° lay-down pattern and fabricated with different filament distances, varying from 1 mm to 2.5 mm, allowing scaffolds with different pore sizes to be produced, as shown in Figure 2.

**Figure 3:**
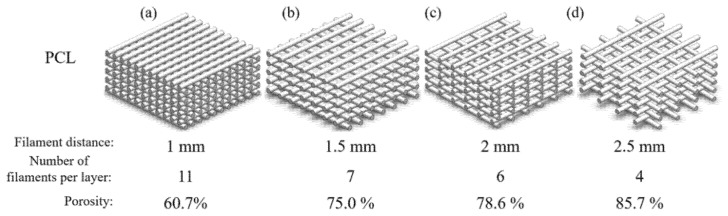
Porosity values as a function of filament distance, (**a**) 1 mm, (**b**) 1.5 mm, (**c**) 2 mm, and (**d**) 2.5 mm for the different designed PCL scaffolds.

The corresponding porosity of the scaffolds was estimated using the following equation, and the results are shown in Figure 3:

(1) $$\mathrm{Porosity}=\frac{\left(F_{\mathrm{dia}}\times N_{l}\times L^{2}\right)-\left[{\left(\frac{F_{\mathrm{dia}}}{2}\right)}^{2}\times \pi \times L\times N_{f}\times N_{l}\right]}{\left(F_{\mathrm{dia}}\times N_{l}\times L^{2}\right)}\times 100$$

where F_dia_ is the filament diameter (mm) of the scaffold, N_l_ is the number of layers, L is the scaffold length/width (mm/mm), and N_f_ is the number of filaments per layer. 

**Figure 4:**
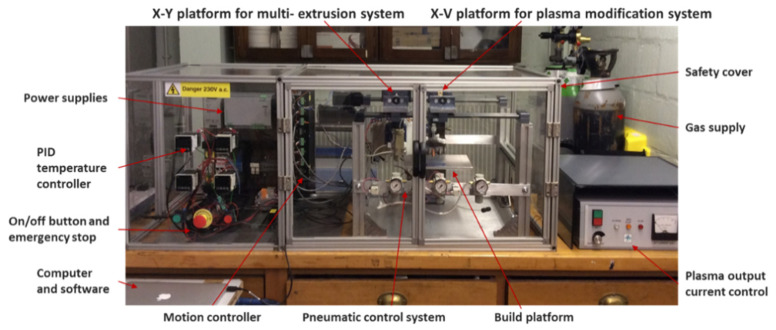
Plasma-assisted bio-extrusion system (PABS) setup.

A novel plasma-assisted bioextrusion system (PABS), being developed by our group at the University of Manchester (Manchester, UK), was used to produce the scaffolds (Figure 4) [15]. The system consists of a multiextrusion unit (two pressure-assisted printing heads and one screw-assisted printing head) and an atmospheric plasma modification unit. This system allows multimaterial scaffolds to be created and the surface modification/coating layer by layer to be selectively performed, enabling the fabrication of functional graded scaffolds. Additional information on this new system can be found in [15,16]. The key processing parameters for scaffold fabrication are presented in Table 1. These processing parameters are the optimised ones, allowing scaffolds with pore sizes and filament diameters similar to the designed models to be obtained.

### 2.3. Mechanical Compression Tests

The compressive modulus of the scaffolds was determined using a single-column table frame INSTRON 3344 (Instron, Norwood, MA, USA) in dry state with a 2-kN load cell and a displacement rate of 1 mm/min, according to the ASTM D695-15. During the compression test, scaffolds were placed between two titanium compression plates and the top plate was attached to the load cell. OriginPro 2019b (Origin Lab Corp, Northampton, MA, USA) was used for data analysis to determine the compressive modulus of the samples as well as for data plots. During compressive tests, the software compiles data of the forces and displacements and converts them into stress and strain values. At least 4 scaffolds were tested for each sample group. Before compressive tests, the machine was calibrated according to the supplier guidelines. The designed dimensions of the different scaffolds are: 5 mm of height and 11 mm of length/width.

### 2.4. Finite Element Simulation

**Figure 5:**
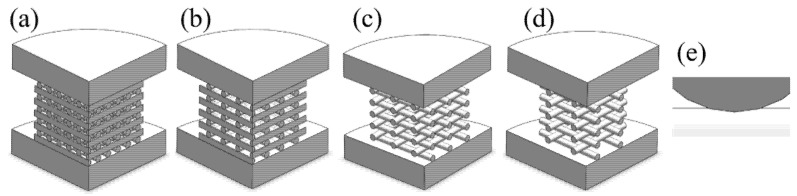
CAD models for the PCL scaffolds with filament distance of (**a**) 1 mm, (**b**) 1.5 mm, (**c**) 2 mm, and (**d**) 2.5 mm, respectively. (**e**) Overlapping of 0.01 mm between two adjacent layers.

Scaffolds were designed in Solidworks, version 2020 (Dassault Systems, Vélizy-Villacoublay, France), and due to their symmetrical geometry, only ¼ of the original models was considered for numerical simulations to reduce the number of elements in the mesh and computational time (Figure 5a–d). Scaffolds were designed considering an overlap of 0.01 mm between each two adjacent layers (Figure 5e). The CAD models were saved as IGS and then imported to the FEA package ANSYS Workbench, version 18.2 (Dassault Systems) for simulation.

The compressive plates were assumed to be made in titanium. A linearly increasing compressive force up to 400 N was applied across the top plate keeping the bottom plate fixed. Moreover, the no separation contact condition was selected for the contact between the filaments and the plates. In the case of no separation contact, sliding is enabled, and the plate/filament gap is eliminated. However, the contacts between each filament were set to bonded in the Ansys software to mimic the actual filaments bonding. A mesh of around 600,000 tetrahedral elements was created, and convergency analysis to achieve this optimised mesh density was conducted.

A static structural analysis system and a linear elastic material property was considered for the prediction of the elastic behaviour of the PCL scaffolds [16]. The Young’s modulus of pure PCL considered in this study is 400 MPa based on previous reported results for the same grade of PCL [16,17]. The Poisson’s ratio was assumed to be 0.442 based on the experimental work conducted by Lu et al. [9].

## 3. Results and Discussion

### 3.1. Mechanical Compression Results

**Figure 6:**
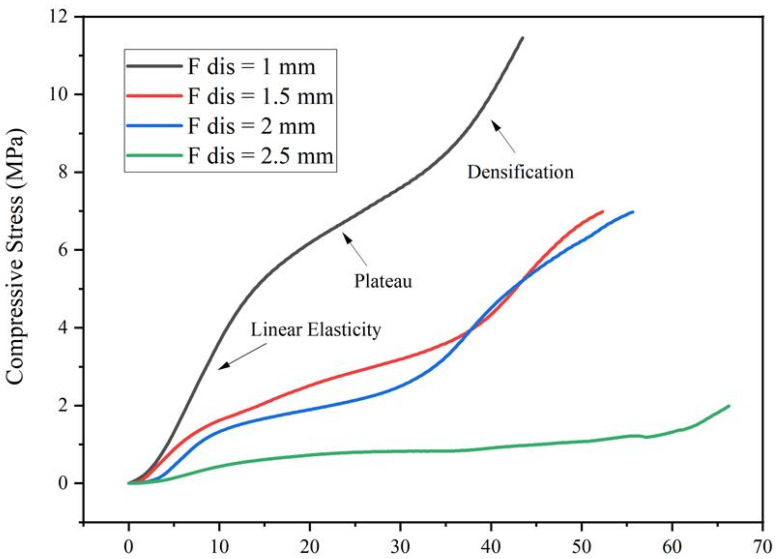
Compressive stress–strain curves for PCL scaffold fabricated with filament distances.

The compressive modulus for each group of scaffolds was evaluated by measuring the compression force that was applied and the corresponding deformation of the scaffolds. Figure 6 shows typical stress–strain curves of printed scaffolds with different pore sizes. The values presented in this figure are the average values obtained from the experimental tests considering four scaffolds per group. These curves show, except for scaffolds designed with 2.5 mm of filament distance, a clear elastoplastic cellular solid behaviour [18]. Three distinguishable regions can be identified: a linear elastic regime, corresponding to the pores edge bending or face stretching; a stress plateau, corresponding to progressive pore collapse by elastic buckling and permanent deformation; and densification, corresponding to the collapse of the pores [19]. Therefore, if not properly designed, the porosity of the scaffolds under compressive loads continuously changes with impact on the scaffold permeability (vascularisation aspects) and cell survival. As experimentally observed, by increasing the pore size, the produced scaffolds exhibit a shorter linear elastic region and a longer plateau, delaying the densification mechanism, which occurs at higher elongation values.

**Figure 7:**
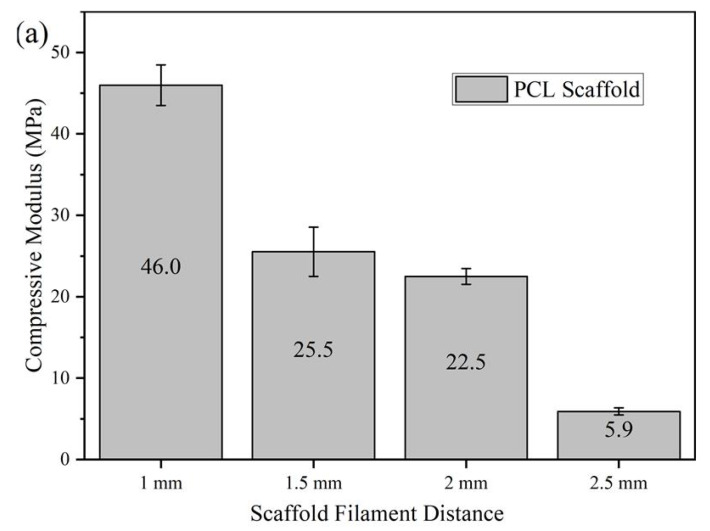

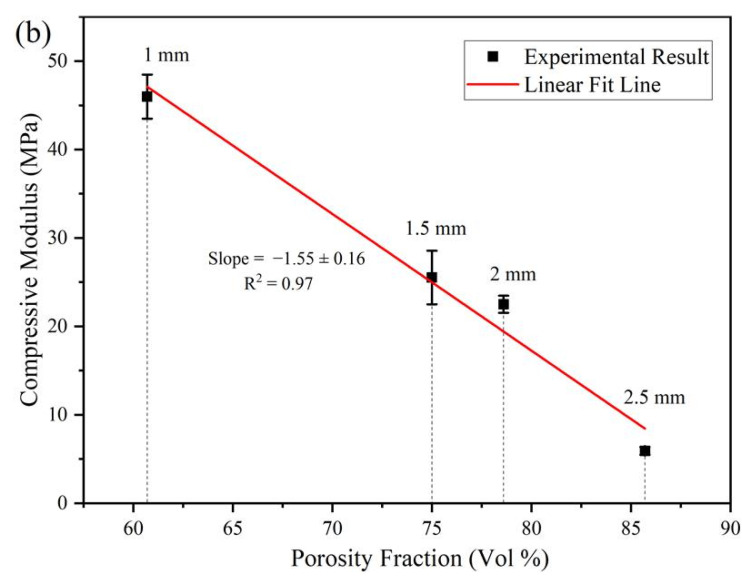
Experimentally obtained compressive modulus. (**a**) Compressive modulus of printed scaffolds considering different filament distances (N = 4). (**b**) The correlation between compressive modulus and the scaffold designed porosity.

The compressive modulus for all produced scaffolds is calculated from the slope of the elastic region in the stress–strain curve, and the variation of the compressive modulus as a function of the scaffold’s initially designed porosity is presented in Figure 7. The compressive moduli were found to be 46.0 ± 2.5 MPa, 25.5 ± 3.0 MPa, 22.5 ± 1.0 MPa, and 5.9 ± 0.4 MPa for scaffolds fabricated with 1 mm, 1.5 mm, 2 mm, and 2.5 mm filament distance, respectively. Moreover, it was possible to observe a linear variation (R^2^ = 0.97) between the compressive modulus and scaffold porosity. The results also show a reduction of the compressive modulus of around 1.55 MPa by increasing the designed porosity by 1%.

### 3.2. Finite Element Analysis Result

**Figure 8:**
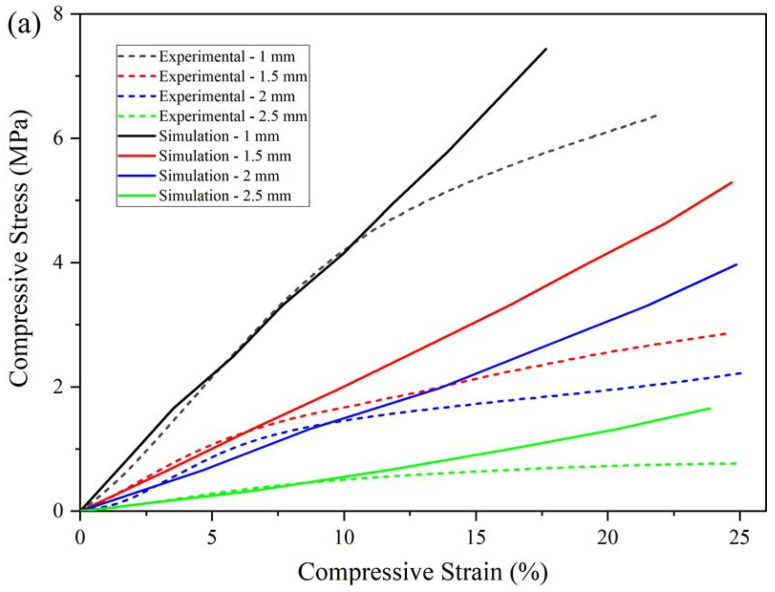

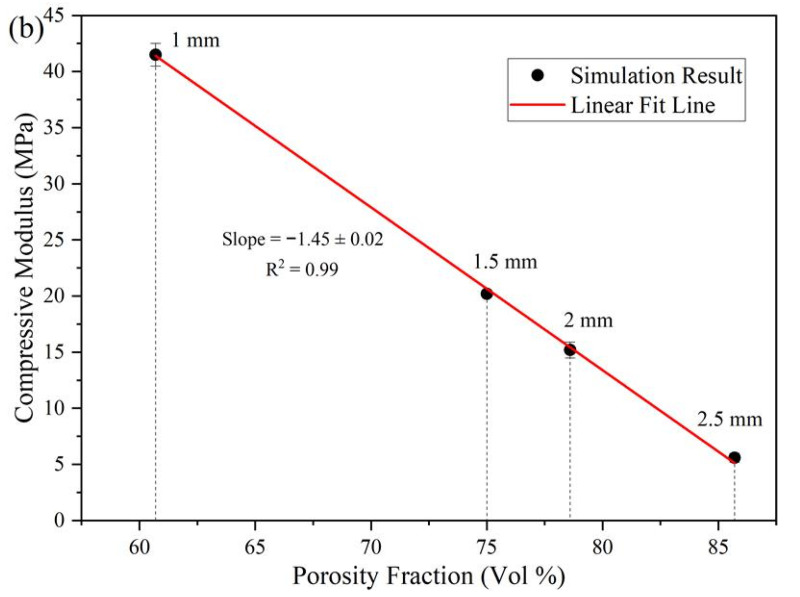
Numerically obtained compressive modulus. (**a**) Compressive modulus of printed scaffolds considering different filament distances (N = 4). (**b**) The correlation between compressive modulus and the scaffold designed porosity.

Simulated stress–strain behaviours of different designed scaffolds are presented in Figure 8a, and the results are plotted against the experimentally obtained curves. As observed, the simulated elastic behaviour of the scaffolds does a good job of describing the physical behaviour of the scaffolds for strain values lower than 10%. Simulations were performed considering the isotropic elastic property model, and consequently, the deformation of the scaffold increases linearly with the increase of compression force. However, once the compression stress exceeds the yield stress, plastic deformation becomes the dominant behaviour, which explains the deviation between experimental and numerical results. Compressive moduli were calculated from the slope of the elastic region in the numerical stress–strain curves, and the variation of the compressive modulus as a function of scaffold porosity is presented in Figure 8b. It was also possible to observe a linear variation (R^2^ = 0.99) between the compressive modulus and scaffold porosity.

Table 2 compares both the numerically and experimentally obtained compressive modulus, making it possible to observe a good level of accuracy of the predicted results. Differences in the results can be attributed to some of our initial assumptions. First, scaffolds were assumed to be homogeneous and isotropic. However, anisotropy is a major issue in additive manufacturing because of the layer-by-layer fabrication approach and the phase change transformation (melting and cooling stages) experienced by the material during the printing process [20]. Using in situ X-ray diffraction (XRD), our group started correlating processing conditions, morphological development, and crystallization, aiming to obtain mathematical models allowing the anisotropy of 3D-printed scaffolds to be designed [20]. Secondly, printed scaffolds usually present pore size values slightly different from the designed ones. This is a result of the solidification of the extruded melted material and the considered processing conditions [21,22]. For example, the filament diameter decreases by increasing the deposition velocity and increases by increasing the screw rotational velocity and processing temperature. Finally, the mechanical properties used in the simulation model are based on the bulk properties of PCL but may not be the same as the grade of PCL used in the experimental work.

**Figure 9:**
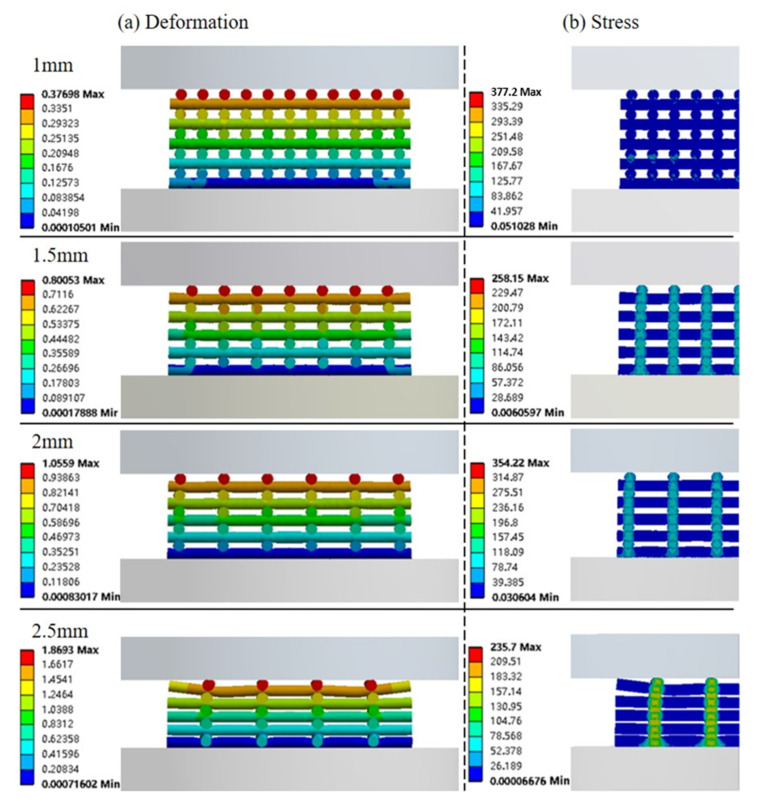
Numerical results showing the (**a**) deformation of the scaffold and (**b**) the stress contours plot for half of the scaffold under a compressive load of 400 N.

Figure 9a shows the deformation of the scaffolds when a compression force of 400 N is applied. Due to differences in porosity, the scaffolds present different mechanical behaviours. As observed, PCL scaffolds designed with 1 mm of filament distance had a maximum deformation of 0.377 mm, while scaffolds designed with a filament distance of 2.5 mm had a maximum deformation of 1.87 mm. Based on these results, it seems that scaffolds designed with a filament distance of 2.5 mm are not suitable for bone applications due to the high deformation that compromises the shape of the pores and, consequently, to the spreading of cells to the internal regions of the scaffold and the supply of oxygen and nutrients. The stress contours plot in Figure 9b (only half of the scaffold is presented) shows that stresses are concentrated along the joint points, with the highest value of 377 MPa being observed for scaffolds with a filament distance of 1 mm. Results also show that the maximum average stress is around 8 MPa (for scaffolds with a filament distance of 1 mm), lower than the compressive yield stress (11 MPa).

## 4. Conclusions

This paper presents a numerical modelling strategy to simulate the mechanical behaviour of scaffolds for bone tissue engineering applications, allowing the compressive modulus and deformation to be calculated. The scaffolds were designed considering regular square pores, different filament distances, and porosities. The scaffolds were printed using an extrusion-based additive manufacturing system and mechanically tested under compressive forces. The results shows a linear decrease of the compressive modulus with porosity (compressive modulus decreases around 1.55 MPa by increasing the porosity by 1%). Despite our initial assumptions, a good agreement between numerical and experimental results (average of around 83%) was achieved. These results suggest that the considered simulation approach is a valid tool for the design of bone scaffolds with a specific compressive modulus, thus reducing the amount of experimental work required for scaffold design. In the future, different pore architectures will be considered to understand the level of sensitivity regarding the topology of the scaffolds. The authors are also planning to further develop the model, correlating the processing conditions and morphological development to capture the effect of material anisotropy. This will require further experimental work to obtain proper mathematical models.

## Figures and Tables

**Table 1:** Printing parameters.

Filament Distance (mm)	1	1.5	2	2.5
Filament diameter (mm)	0.5			
Scaffold length/width (mm)	11			
Number of layers	10			
Deposition velocity (mm/s)	3			
Screw rotation velocity (rpm)	15			
Material chamber temperature (°C)	100			
Screw chamber temperature (°C)	100			

**Table 2:** Comparison between numerically and experimentally obtained compressive modulus.

Filament Distance (mm)	Porosity (%)	Experimentally Obtained Compressive Modulus (MPa)	Numerically Obtained Compressive Modulus (MPa)
1	60.7	46.0 ± 2.5	41.5 ± 1.0
1.5	75.0	25.5 ± 3.0	20.2 ± 0.3
2	78.6	22.5 ± 1.0	15.2 ± 0.7
2.5	85.7	6.0 ± 0.4	5.6 ± 0.4

## Data Availability

The data presented in this study are available on request from the corresponding author.
